# Gut Bacterial 20-Hydroxysteroid Dehydrogenases Modify Endogenous Glucocorticoids and Corticosteroid Drugs

**DOI:** 10.1021/acs.biochem.5c00661

**Published:** 2026-06-24

**Authors:** Sean Coyne, Robert Ghergurovich, Francis Sacco, Francesca Lombardi, Kailey Paar, Jackson DeMartino, Annick A. Kenfack, Tyler M. M. Stack

**Affiliations:** Department of Chemistry and Biochemistry, Providence College, 1 Cunningham Square, Providence, Rhode Island 02918, United States

## Abstract

Bacterial 20-hydroxysteroid dehydrogenases (20-HSDHs) from the human gut are known to reduce the C-20 carbonyl group of cortisol, producing either 20α- or 20β-dihydrocortisol. Two 20-HSDHs from *Bifidobacterium adolescentis* L2–32 and *Agathobaculum desmolans* ATCC 43058, both members of the short-chain dehydrogenases/reductases superfamily, are known to produce 20β-dihydrocortisol, while the 20-HSDH from *Clostridium scindens* ATCC 35704, belonging to the zinc-containing alcohol dehydrogenase family, produces 20α-dihydrocortisol. These three enzymes were characterized for their activity toward cortisol and structurally related therapeutic corticosteroids. Kinetic analyses revealed narrow substrate specificity, with all enzymes preferring cortisol but maintaining significant activity for prednisone and prednisolone (*k*_cat_/*K*_M_ values between 10^3^–10^6^ M^–1^ s^–1^ for the 20β-HSDH enzymes), and some detectable activity of the 20β-HSDHs to reduce triamcinolone. The 20β-HSDHs exhibited pH-dependent substrate inhibition, influencing their activity profile. Structural docking studies indicated that suitable substrates occupy a single productive binding mode within the 20β-HSDH enzyme active site. Our findings show that enzymes in the gut microbiome can metabolize corticosteroid drugs by reducing the 20-keto group, which could have implications for drug efficacy and side effects. This work highlights the importance of gut microbial enzymes in the biotransformation of both endogenous and therapeutic steroids, informing future research into drug-microbiome interactions and personalized medicine.

## Graphical abstract

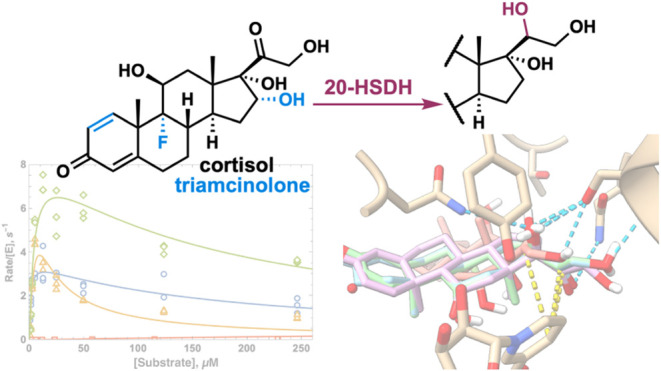

## Introduction

The gut microbiota contributes to human health through diverse metabolic activities, including the biotransformation of dietary compounds and xenobiotics.^1,2^ Given that many genes are uncharacterized or have unknown functions, and that the trillions of organisms that comprise the microbiome contain 150 times as many unique genes as the human host, it remains challenging to predict microbial contributions to human metabolism solely from genetic sequences.^3–6^ Recent research has expanded our understanding of this microbial influence, revealing that gut bacteria can significantly affect the efficacy and pharmacokinetics of various therapeutic agents.^7–10^ Large-scale screening of drug-metabolizing activity has shown that virtually all isolated bacterial strains and gut microbiomes tested exhibit drug metabolic activity, with dozens of drugs found to be structurally altered by gut microbes.^11–13^ These screening efforts identified several gut microbes capable of modifying cortisol, and several cortisol-modifying enzymes have been described *in vitro*.^14,15^ One example of cortisol metabolism is the reduction of the C-20 ketone of cortisol by 20-hydroxysteroid dehydrogenases (20-HSDHs), which perform stereospecific reductions yielding either 20α-dihydrocortisol or 20β-dihydrocortisol by the action of 20α-HSDHs or 20β-HSDHs, respectively (Figure 1a).^14^ The 20β-HSDH enzymes (DesE orthologs) from gut bacteria *Bifidobacterium adolescentis* L2–32 (BaDesE, UniProt ID A7A7R9, GenBank ID WP_003810233.1) and *Agathobaculum desmolans* ATCC 43058 (AdDesE, GenBank ID WP_051643274.1) have been previously characterized and shown to have similar cortisol reductive activities (with 57.8% sequence identity between these two DesE proteins).^16,17^ These 20β-HSDH enzymes belong to the short-chain dehydrogenases/reductases (SDR) superfamily, which also contains the human 11β-HSDH and 17β-HSDH enzymes. A 20α-HSDH enzyme (DesC) from the gut bacterium *Clostridium scindens* ATCC 35704 (CsDesC, UniProt ID B0NC68) catalyzes the reduction of the 20-keto group of cortisol with opposite stereospecificity and instead belongs to the zinc-containing alcohol dehydrogenase family.^18^ These enzymes are reported to have moderate kinetic efficiency with cortisol (*k*_cat_/*K*_m_ values between ∼10^4^–10^6^ M^–1^ s^–1^).^16–18^ Beyond HSDHs, some bacteria, including *C. scindens* ATCC 35704, encode a “desmolase” enzyme (a heterodimeric protein of the transketolase family, DesAB) as part of the same operon as DesC and DesE, capable of removing the two-carbon tail of cortisol and the structurally similar corticosteroid therapeutics such as prednisone, prednisolone, and dexamethasone (Figure 1b).^19–21^ After side-chain cleavage, these products can have potential androgenic activity, leading to the proliferation of cancer cells.^20^ Gnotobiotic mice that are monocolonized by *C. scindens* display side-chain cleavage of the drug dexamethasone *in vivo*, affecting the serum concentration of dexamethasone and its metabolite.^21^ The side-chain cleavage of cortisol and the reduction of the 20-ketone are mutually exclusive metabolic reactions, and an understanding of these two transformations can inform the fate of cortisol metabolites and the potential side effects of cortisol and corticosteroids.

**Figure 1:**
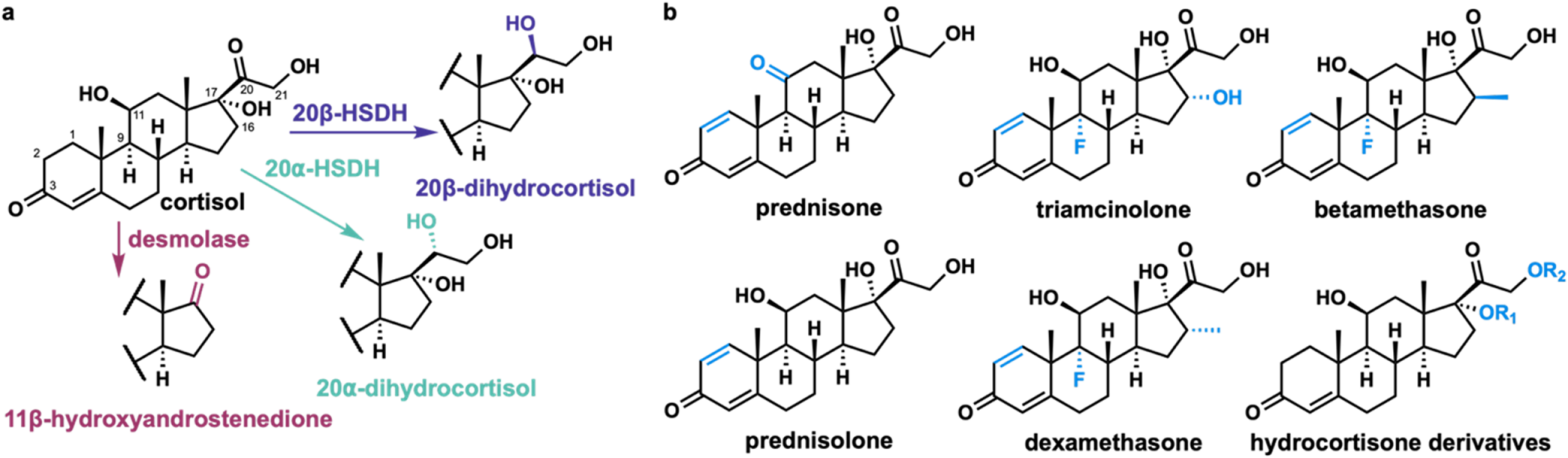
Cortisol and corticosteroid metabolism by human gut microbes. (a) Cortisol is a known substrate for different gut bacterial enzymes, resulting in several different modifications. The steroid numbering referenced in the text is illustrated for cortisol. (b) Comparison of corticosteroid drugs to cortisol, with differences in structure highlighted in blue.

There have been no reports on the activity of 20-HSDHs toward corticosteroids other than cortisol. Additionally, other known glucocorticoid modifications performed by gut microbes include 21-dehydroxylation and full reduction of the A-ring in the OsrABC pathway (forming 3β,5β-tetrahydrosteroid products).^22,23^ Differences in substrate specificity among the DesC and DesE 20-HSDHs, the desmolytic DesAB enzyme, other glucocorticoid modifications like the OsrABC pathway,^23^ and the abundance of these proteins in gut bacteria can lead to different drugs undergoing distinct metabolic pathways, further complicating metabolite prediction in the gut microbiome. Previous studies have found that 40 human gut microbial species encode a DesE homologue, that robust expression of 20β-HSDH genes (and not neighboring genes) is observed in metatranscriptomic data, and that DesC and DesE genes are present on average in 0.6% and 36% of individual microbiomes, respectively, with abundance varying between subjects.^11,15^ This suggests that individual microbiomes can vary in whether these genes are present, but that these cortisol-reducing 20β-HSDH proteins are likely expressed if these genes are present in the bacterial genome.

We sought to utilize the characterized gut-derived cortisol 20-HSDH enzymes, BaDesE, AdDesE, and CsDesC, to determine their specificity for cortisol versus structurally similar therapeutic corticosteroids, and to rationalize substrate specificity using the published structures of BaDesE and CsDesC for virtual substrate docking. Our kinetic analysis of these gut-derived enzymes demonstrated that they exhibit a relatively narrow substrate range, preferring cortisol but showing relatively high activity with the drugs prednisone and prednisolone. An expanded kinetic analysis of the reported maximal rates at different pH values for BaDesE and AdDesE found that these enzymes exhibit pH-dependent substrate inhibition.^16,17^ Docking models of the corticosteroids in the previously reported structure of BaDesE find that suitable substrates occupy a single productive binding mode, while docking in the CsDesC structure was less accurate in its prediction of *in vitro* activity. The substrate scope of these 20-HSDHs, along with their kinetic rates and gut metagenomic abundance, as well as those of the other steroid-modifying enzymes encoded in the gut microbiome, has implications for their roles in drug metabolism and the resulting therapeutic efficacy.

## Materials and Methods

### Materials

Chemicals and biochemicals were purchased from Sigma-Aldrich Chemical Co. and Fisher Scientific, Inc. UV–Visible absorption readings were performed using a Cary 3500 spectrophotometer from Agilent Technologies. Pelleting cells and clarifying cell lysates were performed using a 5910Ri Centrifuge from Eppendorf North America. Codon-optimized genes of the DesE orthologs were prepared (Table S1), cloned into pET28a plasmids by Twist Biosciences, and transformed into XJb (DE3) cells according to the manufacturer’s protocol (Zymo Research). Protein purity was verified by SDS-PAGE using a 4–20% Mini-PROTEAN TGX gel from Bio-Rad Laboratories, Inc. Protein concentration was determined using the theoretical ε_280_ values provided by the ProtParam webtool (https://web.expasy.org/protparam/).

### CsDesC Cloning

*Clostridium scindens* ATCC 35704 was purchased from ATCC and used to inoculate two BHIS agar plates in a Coy Anaerobic chamber. A single colony was used to inoculate 10 mL of BHIS media, and cultures were grown overnight at 37 °C. Genomic DNA was isolated using an NEB Genomic DNA Purification kit, following the manufacturer’s protocols. The CsDesC gene was amplified using Q5 High-Fidelity DNA Polymerase (NEB). Primers were obtained from Integrated DNA Technologies, and the sequences are listed in Table S2. The PCR amplicon was cloned into a pET28a plasmid digested with NdeI and XhoI using Gibson Assembly Master Mix (NEB), following the manufacturer’s protocols.

### Protein Expression

From Luria–Bertani (LB) agar plates, transformed XJb (DE3) cells were inoculated in 5 mL of LB and grown overnight at 37 °C with 30 μg/mL kanamycin. To overexpress the 20-HSDHs, 500 μL of the overnight culture was added to 50 mL sterile ZYM-5052 autoinduction media supplemented with 0.3 mM arabinose and 60 μg/mL kanamycin.^24^ Cultures were grown at 37 °C overnight and then centrifuged at 4000*g* for 15 min at 4 °C. Expression of AdDesE required cold expression, in which cells were initially grown at 37 °C for 4 h, then at 17 °C for 42 h. Cells were resuspended in 5 mL Ni-NTA wash buffer (500 mM NaCl, 50 mM sodium phosphate, 50 mM imidazole, pH 8.0) per 1 g of wet cell weight and stored at −80 °C until purification.

### Protein Purification

Following autoinduction, the frozen cells were incubated in a 37 °C water bath until thawed. When fully thawed, egg white lysozyme (100 mg/mL Gold-Bio), DNase 1 (10 units/mL, Sigma), and 100× ProBlock Gold Bacterial Protease Inhibitor Cocktail (1×, Gold-Bio) were added to a final concentration of 0.5 mg/mL, 10 μL/mL, and 10 μL/mL, respectively. Cells were homogenized using a Benchmark Pulse 150 Ultrasonic Homogenizer with a 10 min cycle time (1 s on, 4 s off). The cell lysate was then centrifuged at 15,000*g* for 45 min at 4 °C and subsequently filtered through a 0.22 μm filter. The clarified lysate was loaded onto a 5 mL HisTrap HP column (Cytiva) equilibrated in Ni-NTA wash buffer on an ÄKTA Go FPLC system (Cytiva). The column was then washed with Ni-NTA wash buffer until protein was no longer detected in the flow-through, and bound protein was eluted in a step gradient using Ni-NTA elution buffer (500 mM NaCl, 50 mM sodium phosphate, 250 mM imidazole, pH 8.0). Fractions containing the desired protein were determined by SDS-PAGE using a 4–20% Mini-PROTEAN TGX gel and purity assessed using ImageJ (Figure S1).^25^ Collected protein samples (approximately 290 mg, 230 mg, 130 mg per 1 L of culture for BaDesE, AdDesE, CsDesC, respectively) were dialyzed overnight at 4 °C in dialysis buffer (200 mM NaCl, 20 mM sodium phosphate, pH 7.2) or the protein buffer was exchanged into dialysis buffer using a PD-10 column according to the manufacturer’s instructions (Cytiva). CsDesC was stored in a buffer containing 0.1 mM ZnSO_4_.

### UV–Visible Substrate Screening of 20-HSDHs

20-HSDH enzymes were screened for activity with cortisol, hydrocortisone-21-acetate, hydrocortisone-17-butyrate, prednisolone, prednisone, fluticasone, fludrocortisone, budesonide, dexamethasone, betamethasone, and triamcinolone. Reaction assays were performed using 200 μM NADH (from a 20 mM stock in buffer), 50 μM substrate (from a 10–30 mM stock in DMSO), and 10 nM of 20-HSDH in 3000 μL 50 mM MOPS pH 7.2 buffer. Enzyme activity was determined by an end point assay. A change in absorbance at 340 nm after overnight incubation, relative to a control assay lacking 20-HSDH, indicates NADH consumption, as measured with a Cary 3500 spectrophotometer (Agilent Technologies).

### ^1^H NMR Analysis of BaDesE and AdDesE Products

^1^H NMR was used to analyze the reduced products of cortisol, prednisone, prednisolone, and triamcinolone to verify the site of reduction (Figures S3–S7). To obtain sufficient product detectable by NMR, the steroid substrate (12 μmol, ∼4.3 mg) was added to a 50 mL round-bottom flask, and buffer (50 mM sodium phosphate, pH 7.2) was added until the substrate was fully dissolved. Then, NADH (18 μmol, 13.2 mg, 1.5 equiv) was dissolved in the steroid solution. An initial absorbance at 340 nm was measured to establish the starting value, and the concentrations of both NADH and the steroid substrate were determined. The reaction was initiated by adding 100 μL of purified DesE (1.0 μM stock), after which the mixture was stirred overnight at room temperature. The reaction progress was monitored by removing aliquots and measuring the absorbance at 340 nm until it reached a constant value, consistent with the calculated absorbance for the expected final NADH concentration (ε_340_ = 6,220 M^–1^ cm^–1^). Reactions with AdDesE achieved ∼10% conversion for the substrates. The steroid products were extracted with ethyl acetate (3 × 20 mL), dried with calcium sulfate, and the solvent was removed. The remaining oil was dissolved in CDCl_3_ and analyzed by ^1^H NMR spectroscopy (Bruker Ascend 400 MHz).

### Enzyme Kinetics

The activities of the 20-HSDHs with their substrates were measured using kinetic assays (performed in triplicate) conducted in 3000 μL of either 50 mM MOPS·NaOH (pH 7.2) or 50 mM sodium acetate (pH 5.0) at room temperature. Kinetic data were determined by a single biological replicate. Upon addition of the 20-HSDH to an assay mixture containing 133 μM NADH and various volumes of substrate dissolved in DMSO, the change in absorbance of NADH over time was measured at each substrate concentration at 340 nm (ε_340_ = 6,220 M^–1^ cm^–1^) in triplicate. The AdDesE and BaDesE enzymes retain full activity up to 4% DMSO at pH 7.2 and 2% DMSO at pH 5.0 (Figure S2). We have limited our kinetic assays to contain less than 1.5% DMSO. The change in absorbance per time was converted to the change in NADH concentration per second and normalized by the enzyme concentration in the assay (rate/[E]). The averages of the technical triplicates at each substrate concentration were fitted to a modified Michaelis–Menten or substrate inhibition equation using Wolfram Mathematica, Version 13.2. The modified Michaelis–Menten equation shown below (eq I) substitutes *K*_m_ with *k*_SP_/*k*_cat_, where the specificity constant, *k*_SP_, is more commonly known as *k*_cat_/*K*_m_.^26,27^ These modified equations are used because they yield the same kinetic constant values while reducing the standard error of the fitted data.^26,27^ *K*_m_ values are then calculated from *k*_cat_ and *k*_SP_.

I $$\frac{v}{\left[E\right]}=\frac{k_{\mathrm{cat}}\left[S\right]}{\left[S\right]+K_{M}}=\frac{k_{\mathrm{cat}}\left[S\right]}{\left[S\right]+\frac{k_{\mathrm{cat}}}{k_{\mathrm{SP}}}}$$

The substrate inhibition eq (eq II) is shown below, with *K*_m_ replaced by *k*_SP_/*k*_cat_.^28^

II $$\frac{v}{\left[E\right]}=\frac{k_{\mathrm{cat}}\left[S\right]}{[S]+K_{M}+\frac{{\left[S\right]}^{2}}{K_{I}}}=\frac{k_{\mathrm{cat}}\left[S\right]}{[S]+\frac{k_{\mathrm{cat}}}{k_{\mathrm{SP}}}+\frac{{\left[S\right]}^{2}}{K_{I}}}$$

In each case, the kinetic constants *k*_cat_ and *k*_SP_ (and *K*_I_ if applicable) are determined by nonlinear data fitting, constrained to positive values, and reported with their standard errors. *K*_M_ values are calculated as *k*_SP_/*k*_cat_, with standard errors determined by error propagation. The inhibition equation was used whenever fitting the enzyme kinetic data yielded smaller standard errors for the determined *k*_SP_ value (Figures S8–S9 and Table S3). The activity of AdDesE with prednisolone at pH 5.0 was best fit with a straight line, where the slope of the line is the *k*_cat_/*K*_m_ value.

### BaDesE Kinetics at pH 7.2

The BaDesE kinetic assay contained 133 μM NADH, 0.00683 μM BaDesE, and varying amounts of substrate solutions (prepared in DMSO) in 3000 μL assays with 50 mM MOPS at pH 7.2. Cortisol, prednisone, prednisolone, and triamcinolone were tested at concentrations ranging from 0.55 to 500 μM. Triamcinolone was tested with 0.309 μM BaDesE. Assays were monitored for 10 min.

### BaDesE Kinetics at pH 5.0

The BaDesE kinetic assay contained 133 μM NADH and varying amounts of substrate solutions (prepared in DMSO) in 3000 μL assays with 50 mM sodium acetate buffer at pH 5.0. Cortisol, prednisone, and prednisolone were tested with assay concentrations ranging from 4 to 250 μM, with 0.35 μM BaDesE. Assays were monitored for 1 min.

### AdDesE Kinetics at pH 7.2

The AdDesE kinetic assay contained 133 μM NADH and varying amounts of substrate solutions (prepared in DMSO) in 3000 μL assays with 50 mM MOPS buffer at pH 7.2. Cortisol, prednisone, and prednisolone were tested with assay concentrations ranging from 4 to 250 μM, using 0.157, 0.239, and 0.223 μM AdDesE, respectively. Triamcinolone was tested from 0.25 to 250 μM with 0.438 μM AdDesE. Most assays were monitored for 1 min, except triamcinolone assays were monitored for 10 min.

### AdDesE Kinetics at pH 5.0

The AdDesE kinetic assay contained 133 μM NADH and varying amounts of substrate solutions (prepared in DMSO) in 3000 μL assays with 50 mM sodium acetate buffer at pH 5.0. Cortisol, prednisone, and prednisolone were tested at pH 5.0 in concentrations ranging from 4 to 250 μM, with 0.266 μM AdDesE for cortisol and prednisone, and 0.55 μM AdDesE for prednisolone. Assays were monitored for 1 min.

### CsDesC Kinetics

The CsDesC kinetic assays contained 133 μM NADH in 3000 μL assays with 50 mM MOPS buffer at pH 7.2. Prednisolone was tested in duplicate, using concentrations ranging from 25 to 350 μM with 0.48 μM CsDesC. Assays were monitored for 1 min.

### Modeling

Computational docking of 20-HSDHs and potential substrates was performed using AutoDock Vina.^29,30^ A model of BaDesE was generated using a single monomer from PDB 6OW4, which contains a bound NAD^+^ in the active site. The structure was determined with the catalytically inactive Ser181Ala mutation, and this mutation was reverted *in silico* for use as the target for docking. The molecules used for docking were uploaded using their SMILES strings from the PubChem database. A model of CsDesC with NAD^+^ in the active site was generated by aligning CsDesC, PDB 4OH1, with the structure of l-threonine 3-dehydrogenase from *Pyrococcus horikoshii* ATCC 700860 containing a bound NAD^+^, PDB 2D8A, and then removing the remaining atoms from 2D8A, leaving an approximate model for NAD^+^ binding to 4OH1.

## Results and Discussion

### Purification and Kinetic Characterization of 20-HSDHs

The 20-HSDH enzymes BaDesE, AdDesE, and CsDesC, which have previously been reported to catalyze the NADH-dependent reduction of cortisol, were overexpressed in XJb (DE3) cells and purified to near homogeneity (≥98%) with the DesE homologues and 85% purity with CsDesC (Figure S1). We found consistent specific activity levels for these three purified enzymes with the reported values.^16–18^ We verified that the products of the BaDesE- and AdDesE-catalyzed reduction of cortisol, prednisone, prednisolone, and triamcinolone contain C20-hydroxyl groups by ^1^H NMR analysis (Figures S3–S7). The hydroxymethylene signals corresponding to the protons on C21 (a pair of doublets found between ∂ 4.0–4.6 ppm) are unique in these structures and diagnostic of the site-specific reduction by DesE. After reduction, these peaks shifted, and new peaks corresponding to both the C20 proton and the two C21 protons appear (between ∂ 3.7–3.8 ppm).

The previous report characterizing BaDesE indicated that it has a maximum rate at pH 5.0, so several FDA-approved corticosteroids were first screened for activity at pH 7.2 and then again at pH 5.0.^17^ The steady-state kinetic constants of BaDesE at both pH values are shown in Table 1, and corresponding fits to the kinetic data are shown in Figure 2. At pH 5.0, BaDesE demonstrates effective reduction of cortisol, prednisone, and prednisolone (Figure 2a), with no detectable activity with the other tested substrates (dexamethasone, betamethasone, triamcinolone, fludrocortisone, hydrocortisone 17-butyrate, and hydrocortisone 21-acetate). It was previously reported that BaDesE displays ∼10% of its activity at pH 7 with 50 μM cortisol compared with pH 5, and we observe a similar ratio when measuring specific activity with 50 μM cortisol.^17^ Our kinetic analysis, however, finds that the *k*_cat_/*K*_m_ value with cortisol is higher at pH 7.2 than at pH 5.0 (a 29-fold increase), but due to substrate inhibition at pH 7.2, the overall rate is depressed at concentrations of 50 μM or above (Figure 2b). The substrate inhibition observed only at pH 7.2 can explain the previously reported maximal rate of BaDesE at pH 5.0, which was reported at a single concentration.^17^

**Figure 2:**
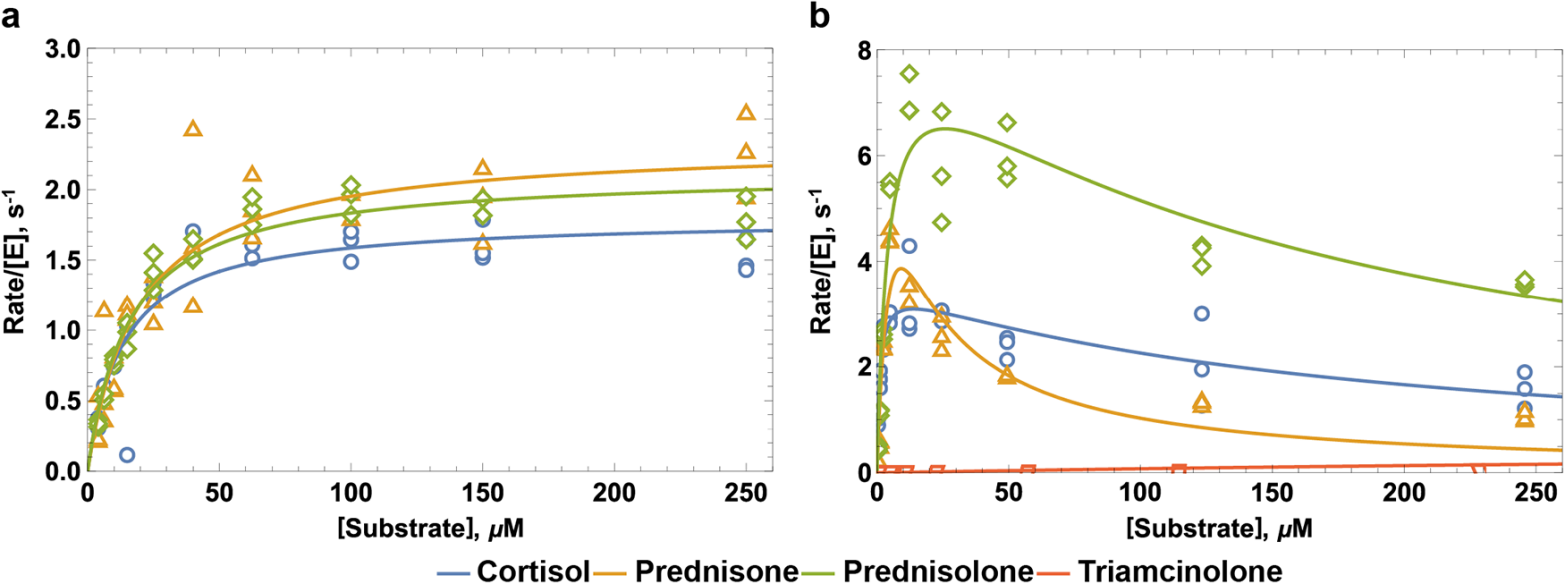
Comparison of the kinetic data of BaDesE with various substrates at (a) pH 5.0, without substrate inhibition, and (b) pH 7.2, with substrate inhibition.

**Table 1:** Kinetic Parameters of BaDesE with Indicated Substrates at pH 5.0 and pH 7.2^a^

Substrate	pH	*k*_cat_ (s^–1^)	*k*_cat_/*K*_m_ (M^–1^ s^–1^)	*K*_m_ (μM)	*K*_I_ (μM)
Cortisol	5.0	1.8 ± 0.1	(1.4 ± 0.2) × 10^5^	13 ± 3	N.A.
Prednisone	5.0	2.3 ± 0.1	(1.2 ± 0.1) × 10^5^	19 ± 2	N.A.
Prednisolone	5.0	2.12 ± 0.09	(1.3 ± 0.2) × 10^5^	16 ± 2	N.A.
Cortisol	7.2	3.6 ± 0.2	(3.2 ± 0.5) × 10^6^	1.1 ± 0.2	170 ± 30
Prednisone	7.2	10. ± 8	(1.4 ± 0.6) × 10^6^	7 ± 7	10 ± 10
Prednisolone	7.2	9 ± 1	(2.0 ± 0.6) × 10^6^	4 ± 1	160 ± 70
Triamcinolone	7.2	0.58 ± 0.03	(8.9 ± 0.2) × 10^2^	650 ± 40	N.A.

a N.A. not applicable.

We observed that the *k*_cat_/*K*_m_ values of the BaDesE-catalyzed reduction of cortisol, prednisone, and prednisolone increased ∼10-fold when the pH was raised from 5.0 to 7.2, primarily due to a decrease in *K*_m_, with a minor increase in *k*_cat_. We observe substrate inhibition for each substrate only at pH 7.2, with *K*_I_ values 155- and 40-fold greater than their respective *K*_m_ values for cortisol and prednisolone, respectively. Interestingly, we find effectively equivalent *K*_m_ and *K*_I_ values with prednisone, resulting in a steeper decrease in activity at higher concentrations than cortisol or prednisolone. Significantly reduced activity is observed with triamcinolone, which does not appear to saturate or exhibit substrate inhibition at our highest tested concentration (250 μM), resulting in a ∼3500-fold decrease in *k*_cat_/*K*_m_. We did not detect any reduction of triamcinolone by BaDesE at pH 5.0.

The similar AdDesE enzyme (with 57.8% sequence identity to BaDesE) was also screened for activity and displayed an identical substrate preference profile. Unlike BaDesE, the previous study that characterized AdDesE reported that it has a maximal rate at pH 7, and displays ∼75% of that rate at pH 5.^16^ We again see substrate inhibition at pH 7.2, as with BaDesE. The kinetic parameters of AdDesE are listed in Table 2, and the fits of the kinetic data are shown in Figure 3. AdDesE demonstrates a similar profile to BaDesE; AdDesE demonstrates efficient reduction of only cortisol, prednisone, and prednisolone at pH 5.0, and while only displaying substrate inhibition at pH 7.2, and additionally showing nonsaturating and lower levels of activity with triamcinolone.

**Figure 3:**
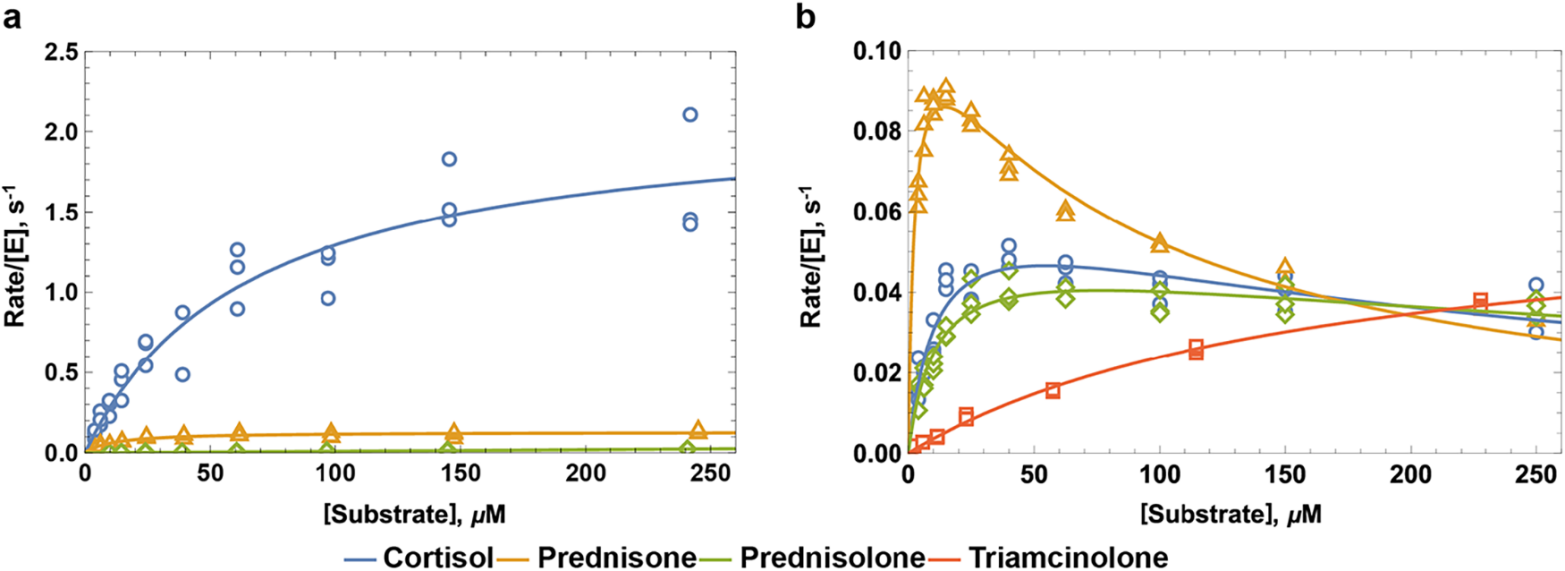
Comparison of the kinetic data of AdDesE with various substrates at (a) pH 5.0, without substrate inhibition, and (b) pH 7.2, with substrate inhibition.

**Table 2:** Kinetic Parameters of AdDesE with Indicated Substrates at pH 5.0 and pH 7.2^a^,^b^

Substrate	pH	*k*_cat_ (s^–1^)	*k*_cat_/*K*_m_ (M^–1^ s^–1^)	*K*_m_ (μM)	*K*_I_ (μM)
Cortisol	5.0	2.1 ± 0.2	(3.3 ± 0.4) × 10^4^	65 ± 9	N.A.
Prednisone	5.0	0.129 ± 0.005	(1.1 ± 0.1) × 10^4^	12 ± 2	N.A.
Prednisolone	5.0	N.D.	(8.9 ± 0.5) × 10^1^	>250 μM	N.A.
Cortisol	7.2	0.065 ± 0.008	(6 ± 1) × 10^3^	11 ± 2	300 ± 100
Prednisone	7.2	0.116 ± 0.009	(5 ± 1) × 10^4^	2.5 ± 0.6	80 ± 20
Prednisolone	7.2	0.052 ± 0.004	(4.8 ± 0.7) × 10^3^	11 ± 2	500 ± 200
Triamcinolone	7.2	0.063 ± 0.005	(3.9 ± 0.3) × 10^2^	160 ± 20	N.A.

a N.A. not applicable.

b N.D. not determined.

The *k*_cat_/*K*_m_ values of AdDesE at pH 7.2 are virtually indistinguishable between cortisol and prednisolone, with a 10-fold increase with prednisone and a 15-fold decrease in value with triamcinolone. Comparison of the AdDesE and BaDesE kinetic values shows markedly decreased *k*_cat_ values with AdDesE, showing a 55-, 86-, 167-, and 9-fold reduction in *k*_cat_ for cortisol, prednisone, prednisolone, and triamcinolone, respectively, compared to BaDesE at pH 7.2. Substrate inhibition with AdDesE is like that of BaDesE, with cortisol and prednisolone showing *K*_I_/*K*_m_ ratios of 27 and 45, respectively. Prednisone again exhibits the greatest substrate inhibition of AdDesE, but at higher concentrations than with BaDesE (*K*_I_ values of 80 μM and 10 μM for AdDesE and BaDesE, respectively).

At pH 5.0, the *k*_cat_/*K*_m_ value is highest for cortisol and 3-fold lower for prednisone. At this pH, prednisolone shows a steep loss of activity, with a *k*_cat_/*K*_m_ value 370-fold lower than that of cortisol, which does not saturate under the same conditions (*K*_m_ > 250 μM). Except with prednisolone, the *k*_cat_/*K*_m_ values are similar to those obtained at pH 7.2, with a 5-fold increase with cortisol (because of a 30-fold increase in *k*_cat_ and a smaller increase in *K*_m_) and a 5-fold decrease with prednisone at pH 5.0 (because of a 5-fold increase in *K*_m_). Compared to BaDesE at the same pH, AdDesE demonstrates only a small reduction in *k*_cat_/*K*_m_ values with cortisol and prednisone (4-fold and 11-fold, respectively), but a nearly 1500-fold decrease with prednisolone.

We initially sought to characterize both BaDesE and AdDesE because, although they share 57.8% sequence identity, they have been reported to have different pH preferences.^16,17^ AdDesE was reported to have maximum activity between pH 7.0 and 7.5, while BaDesE instead showed greatest activity at pH 5.0. Although a physiological hypothesis for this change in pH maximum was not provided, we sought to determine whether these enzymes exhibit different maximal rates across those two pH values. We have found that these enzymes exhibit the same pH-dependent substrate inhibition and relative substrate preferences. Our observation of substrate inhibition at pH 7.2 but not at pH 5.0 explains the differences in specific activity reported for BaDesE and highlights the benefit of our kinetic analysis in identifying this phenomenon.^17^ A previous report tested whether a change in pH from 6.0 to 8.0 could alter the activity of DesE homologues capable of reducing hydrocortisone (cortisol) and other xenobiotics in an end point assay and found no change in conversion.^15^ Given that *Escherichia coli* maintains a cytoplasmic pH between 7.2 and 7.8, and bacterial cytosolic pH is generally between 6.0 and 8.0, and luminal pH in the colon rarely drops below 5.5, we would expect the 20β-HSDH enzymes to display substrate inhibition in their native environment.^31–33^

Measurements of prednisolone and dexamethasone in germ-free mice, administered as a 10 mg/kg and 2 mg/kg gavage, respectively, showed that the concentration in the colon reached about 0.2–1 pmol/mg (estimated to be ∼0.25–1 μM).^21,23^ In that case, substrate-inhibition may not be apparent physiologically unless an enteric formulation of the corticosteroids is administered. An analysis of the concentrations of dexamethasone and its side-chain cleavage product by DesAB in gnotobiotic mice monocolonized with *Clostridium scindens* showed increased concentrations of this metabolite in both the large intestine and serum.^21^ In a similar study, the measurement of the reductive metabolites of prednisolone produced by OsrABC in gnotobiotic mice monocolonized with *Clostridium steroidoreducens* were measured at concentrations around 0.1 pmol/mg in the colon.^23^ As previous studies observed 20-HSDH activity with cortisol in *ex vivo* incubations of human gut microbiomes, 20-HSDH enzymes should be further investigated in future *in vivo* studies for metabolite production for their potential contribution in drug metabolism.

CsDesC catalyzes a similar reduction as BaDesE and AdDesE, but instead belongs to a zinc-dependent family of dehydrogenases and performs the opposite stereochemical reduction, resulting in the formation of 20α-hydroxysteroids.^18^ Screening CsDesC for substrates at pH 7.2 yielded the same set of accepted substrates as BaDesE and AdDesE in a simple end point assay using cortisol, prednisone, prednisolone, and triamcinolone. In our hands, CsDesC lost activity within 24 h, making it difficult to obtain a full kinetic characterization of all substrates. We were able to perform kinetic trials in duplicate with CsDesC and prednisolone at pH 7.2, and fitting the data yields kinetic constants of *k*_cat_/*K*_m_ = (1.8 ± 0.4) × 10^3^ M^–1^ s^–1^, *k*_cat_ = 0.28 ± 0.05 s^–1^, and *K*_m_ = 160 ± 40 μM (Figure S10). A previous study reported that this enzyme catalyzes the NADH-dependent reduction of cortisol with kinetic constants of *k*_cat_/*K*_m_ = 1.4 × 10^4^ M^–1^ s^–1^, *k*_cat_ = 0.02 s^–1^, and *K*_m_ = 1.46 μM.^18^ This is a lower turnover with cortisol than the 20β-HSDH enzymes, but does not rule out its contribution to drug metabolism *in vivo*.

The 20β-HSDH enzymes display near-equivalent reduction efficiencies of prednisone and prednisolone compared with the native substrate, cortisol (except AdDesE at pH 5, which does not efficiently reduce prednisolone). This finding indicates that there are relatively few modifications to the core structure of cortisol that can still bind productively to these enzymes (although one report found that DesE homologues can reduce the ketones of nonsteroid drugs, such as nabumetone, and the aglycone metabolite of doxorubicin, in an end point assay).^15^ Prednisolone differs from cortisol only by the addition of a double bond between carbons 1 and 2, while prednisone has this same Δ^1^ modification and bears an oxidation of the 11β-hydroxy group to a ketone. These modifications are comparatively distal from C20, the site of reduction, and as such, do not alter any kinetic constant by more than 10-fold relative to cortisol. Previous studies have found that BaDesE and AdDesE show a 55% and 50% reduction in specific activity with 50 μM 3α,5β-tetrahydrocortisol (a reduction of the C4–C5 double bond and the 3-keto group of cortisol, a diastereomer of the OsrABC product), respectively, demonstrating that structural changes to the A-ring of the steroid substrate show similar reduction of activity, whether it is the removal or addition of unsaturation.^16,17,23^ Given the similar kinetic parameters generally observed for prednisone and prednisolone, modification of the 11-hydroxyl group to a ketone has little effect, and is further supported by previous reports that these enzymes demonstrate a 34% and 50% reduction in specific activity with 11-deoxycortisol (BaDesE and AdDesE, respectively) compared to cortisol.^16,17^ Adding ester groups to the C21 or C17 hydroxyl groups precludes activity with these enzymes, demonstrating that modifications closer to the reaction site are not permitted. Triamcinolone bears a 9α-fluoro and 16α-hydroxy group on the prednisolone structure, and this leads to a near 100-fold reduction in the *k*_cat_/*K*_m_ values in triamcinolone compared to prednisone. There is no observed activity with dexamethasone, which replaces the 16α-hydroxy group of triamcinolone with a 16α-methyl group, indicating that only a polar substituent can be accommodated at this position, albeit poorly. We could not detect any activity of these enzymes with betamethasone, the 16-epimer of dexamethasone.

We next sought to model cortisol and the other tested steroid substrates within the active sites of these 20-HSDH enzymes using AutoDock Vina to determine whether their relative activity and stereochemistry could be computationally predicted. If successful, then future molecules could be virtually screened to determine if 20-HSDH metabolism is possible. Our proposed catalytic mechanism for BaDesE and AdDesE follows those found in the SDR superfamily (Figure 4a).^34^ NADH first binds, followed by cortisol, and Ser181 (using BaDesE numbering) forms a hydrogen bond with the C20 carbonyl oxygen to facilitate the hydride transfer from NADH. Tyr200 acts as the general acid, donating a proton from the phenolic hydroxy group to the newly formed 20β-hydroxyl group, with the nearby Lys204 lowering the p*K*_a_ of Tyr200 to facilitate this role. The structure of BaDesE (PDB 6OW4) has a bound NAD^+^, with the inactivating mutation S181A. We used this structure, changing the alanine back to the critical active site Ser181 residue *in silico*, and used AutoDock Vina to model cortisol and other steroids binding to the active site (results shown in Table 3). We found that the preferred substrates (cortisol, prednisone, and prednisolone) could be modeled in the active site of BaDesE, with the C20 carbon of the steroidal substrates positioned 3.8 Å from the site of hydride transfer, specifically at C4 of the nicotinamide of NAD^+^ (Figure 4b). This productive binding orientation modeled between the reactive sites would produce a 20β-hydroxy product, as observed for this enzyme, delivering the 4-pro-*S* hydride from NADH, as seen in other enzymes in the SDR superfamily.^34^ In our docking model, we see the noncatalytic Ser183 side chain within hydrogen bonding distance of both hydroxyls on C17 and C21 of the substrates, along with the Asn132 side chain and the Ser183 peptidic nitrogen within hydrogen bonding distance with the C17 and the C21 hydroxyl groups, respectively. The C17 and C21 esters of cortisol are not substrates for BaDesE, and an analysis of the cortisol binding mode shows there is little space to accommodate any substituents at these positions (Figure 4c). Triamcinolone, dexamethasone, and betamethasone bear an additional substituent on C16, and these all result in nonproductive binding modes, although a lower-scored binding mode for triamcinolone is observed, with the distance between the reacting carbon atoms having increased to 5.1 Å (Figure 4b). Together, the computational docking results are consistent with the predicted mechanism based on the well-characterized SDR superfamily, and support the similar kinetic values observed for cortisol, prednisone, and prednisolone, and provide some rationale for the lowered activity of triamcinolone, as well as the lack of observed activity with dexamethasone, betamethasone, and the cortisol esters at C17 or C21.^34^

**Figure 4:**
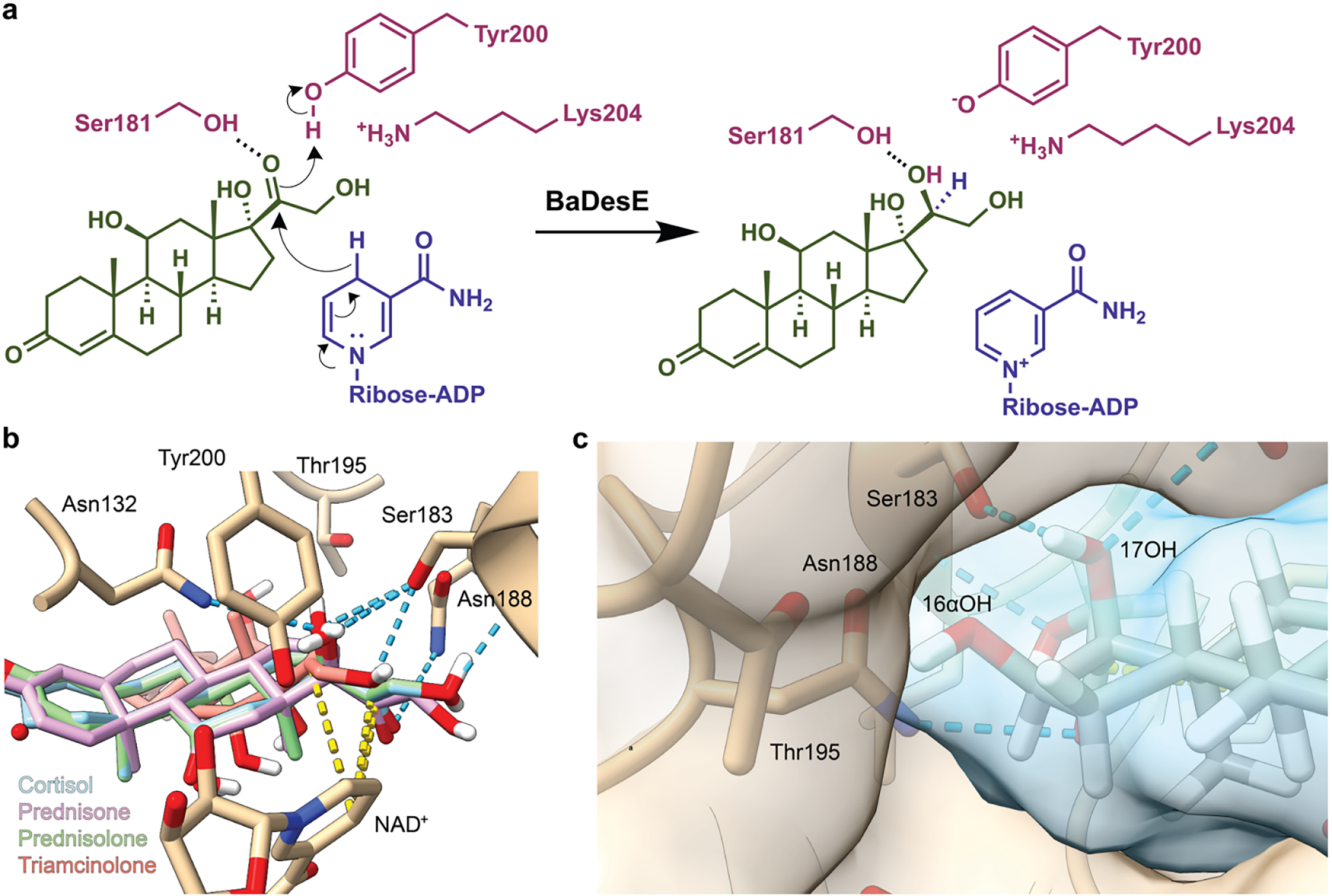
BaDesE mechanism and docking analysis with cortisol and other corticosteroids. (a) The proposed mechanism of BaDesE, given other members in the SDR superfamily, involves the NADH-dependent reduction of cortisol using important active site residues Ser-181, Tyr-200, and. Lys-204. (b) Docking model of cortisol, prednisone, prednisolone, and triamcinolone to BaDesE (PDB 6OW4, with NAD^+^ bound). Yellow dashed lines indicate the distance between the C20 carbonyl of the substrates and the C4 of NAD^+^. Potential hydrogen bond interactions are shown with blue dotted lines. (c) The active site of BaDesE is closely fit around the hydroxyl groups on C17 and C21 of cortisol. Placing a 16α-hydroxy group on cortisol, as in triamcinolone, results in steric clashes with Asn188 and Thr195.

**Table 3:** AutoDock Results between BaDesE (PDB ID 6OW4) and Various Substrates

Substrate	Binding Energy (kcal/mol)	C20 (steroid)–C4 (NAD) distance	Binding orientation	BaDesE substrate?
Cortisol	–8.108	3.8 Å	Productive	Yes
Cortisol-17-butyrate	–6.647	7.4 Å	Nonproductive	No
Cortisol-21-acetate	–7.015	7.0 Å	Nonproductive	No
Prednisone	–7.852	3.8 Å	Productive	Yes
Prednisolone	–7.736	3.8 Å	Productive	Yes
Triamcinolone	–7.148	3.6 Å	Nonproductive	Yes
	–6.852	5.1 Å	Productive	
Dexamethasone	–7.549	3.7 Å	Nonproductive	No
Betamethasone	–7.884	3.6 Å	Nonproductive	No

Similar docking analysis with CsDesC was less informative than with BaDesE. The binding energies of the docked molecules and the distances between reactive centers are listed in Table 4. Unlike BaDesE, the docking data for CsDesC do not align with our end point assays and yield lower calculated binding energies, indicating that a preliminary virtual binding screen does not correlate with experimental findings. Intriguingly, for every molecule tested, some higher-scoring models placed the substrates in positions to react with the C3 carbonyl, or to reduce the C20 carbonyl with the incorrect stereochemistry.

**Table 4:** AutoDock Results between CsDesC (PDB ID 4OH1) and Various Substrates

Substrate	Binding Energy (kcal/mol)	C20 (steroid)–C4 (NAD) distance	Binding orientation	CsDesC substrate?
Cortisol	–6.300	3.8 Å	Productive	Yes
Cortisol-17-butyrate	N.D.	N.D.	Nonproductive	No
Cortisol-21-acetate	N.D.	N.D.	Nonproductive	No
Prednisone	–6.430	4.0 Å	Productive	Yes
Prednisolone	–5.893	4.1 Å	Productive	Yes
Triamcinolone	–6.340	3.7 Å	Productive	Yes
Dexamethasone	–6.648	3.7 Å	Productive	No
Betamethasone	–6.708	4.0 Å	Productive	No

The docking experiments do not provide any immediate hypotheses for our observed pH-dependent substrate inhibition. The only ionizable groups in the active site are those in the Ser/Tyr/Lys catalytic triad. It has been reported that the Tyr/Lys of the *Drosophila lebanonensis* alcohol dehydrogenase of the SDR family acts as a single titratable unit with a p*K*_a_ of 7.2, and that the overall charge changes from 0.50 at pH 7.2 to about 0.65 at pH 5.0.^35^ This relatively small change in the protonation state of the active-site residue is unlikely to cause substrate inhibition. Substrate inhibition as a function of pH was explored for the *Mycobacterium tuberculosis* 3-phosphoglycerate dehydrogenase enzyme (not in the SDR superfamily, with 22.4% identical residues to BaDesE).^36^ They found a domain containing an “anionic binding site” in a separate domain from the active site, and the cationic residues that make up this separate site are the source of pH-dependent substrate inhibition. There is no equivalent domain in the DesE enzymes. In another case, the human serine hydroxymethyltransferase enzyme binds its two substrates, serine and tetrahydrofolate (THF), in random order, and pH-dependent substrate inhibition arises from a dead-end binding complex between the substrate THF and the product, glycine, at lower pH.^37^ Such a mechanism is possible for the DesE enzymes, which should follow an ordered bibi mechanism.^34^ In a normal cycle, NADH binds, followed by cortisol binding, then after the reaction, 20β-dihydrocortisol is released, and finally NAD^+^ is released. If the dead-end ternary complex with NAD^+^ and cortisol binds with greater affinity at pH 7.2 than at pH 5.0, this could explain the substrate inhibition, albeit with an unclear mechanism for how changes in ionic binding would occur between DesE and cortisol. Other routes could involve changes in the ionization state of DesE, most likely involving histidine residues, which should be fully ionized at pH 5.0 and neutral at pH 7.2, although there are no candidate residues near the active site. The results presented here do not suggest a potential mechanism; as such, the basis of pH-dependent substrate inhibition remains undetermined.

Our results demonstrate that the reduction of corticosteroids to produce their corresponding 20-hydroxy metabolites should occur alongside cortisol reduction in the human gut, although these metabolites have not been reported to cause any beneficial or deleterious effects.^11,12^ It has been reported that reductions in the A-ring of prednisolone by OsrABC produce 3β,5β-tetrahydrocortisol, which can reduce serum levels of prednisolone in mice, and that the genes encoding these proteins are found in higher abundance in the metagenomes of Crohn’s disease patients.^23^ As such, microbiome-derived metabolites of corticosteroid drugs have clinical implications for their use in treating inflammatory intestinal diseases.

These 20-HSDH enzymes, DesC and DesE, and the desmolase enzyme, DesAB, are encoded on the same operon and utilize cortisol as a substrate.^14^ Given that the DesAB enzyme from the gut microbe *C. scindens* ATCC 35704 has been shown to convert corticosteroid drugs into androgenic compounds, others have hypothesized that having highly active 20-HSDH enzymes may protect the host from the formation of these metabolites, a concern for diseases such as castration-resistant prostate cancer or polycystic ovary syndrome, and clinical implications for postmenopausal women.^14,19,20,38^ We noted that C17- or C21-esters of cortisol prevent the activities of these enzymes, but ester hydrolysis by gut microbes, coupled with C20 reduction of cortisol, has been previously observed.^11^ As such, the role of 20-HSDHs in preventing the formation of androgenic compounds from corticosteroid drugs in the gut (and other steroid-modifying enzymes, like the 3-oxo- or Δ^4^-reductases) should be further investigated to see if these enzymes may alleviate these concerns.^23,39,40^ Some therapeutics, such as dexamethasone, cannot be reduced by 20-HSDHs but can be substrates for both DesAB and those in the OsrABC pathway.^20,23^

Together, we have provided *in vitro* characterization of the gut-derived 20-HSDH enzymes and found that they have narrow specificity but are relatively efficient at the non-native steroidal drugs prednisone and prednisolone. The previous observation that the AdDesE and BaDesE enzymes exhibit maximum activity at different pH values may have been obscured by pH-dependent substrate inhibition, as determined by our kinetic characterization. Given the relatively low steroid concentrations expected in the lower intestines, this inhibition may not be consequential *in vivo*.^21,23^ Our analysis of the CsDesC enzyme is limited, but we observed NADH-dependent reduction of corticosteroid drugs in a simple end-point assay, and that our computational modeling was uninformative. However, we demonstrate that computational docking of the more abundant and kinetically efficient DesE 20β-HSDH enzymes is generally predictive of their relative activity and can be used to predict whether other steroids may also be modified by the gut microbiome.

## Conclusions

In summary, our investigation has demonstrated the efficient reduction of corticosteroids by microbial 20-hydroxysteroid dehydrogenases found within the human gut. By leveraging computational docking approaches, we found that the docking results were consistent for the steroids we tested with 20β-HSDH (but not 20α-HSDH) and may provide a useful basis for evaluating additional, as-yet untested drugs. The structural diversity of these therapeutics, together with the specificity and abundance of microbial enzymes (and genes that encode them), may shape their metabolic outcomes and ultimately influence therapeutic efficacy and safety profiles. These findings inform future studies of the interplay between drug structures, the gut microbiota, and host physiology, advancing our understanding of drug-microbe interactions at the molecular level.

## Supplementary Material



## Notes

Codon-optimized DNA sequences used for expression, primers used to clone the CsDesC gene, the aligned ^1^H NMR spectra of the BaDesE and AdDesE products, and further kinetic analysis of the 20-HSDH enzymes (PDF)
