# Supplementary material for: Gut Bacterial 20-Hydroxysteroid Dehydrogenases Modify Endogenous Glucocorticoids and Corticosteroid Drugs
Source: Biochemistry. 2026 Jun 24;65(13):2180–9. doi: 10.1021/acs.biochem.5c00661 (PMC13348027; doi:10.1021/acs.biochem.5c00661)
Supplement: Supplementary file 1 [file bi5c00661_si_001.pdf]

“Gut Bacterial 20-Hydroxysteroid Dehydrogenases Modify Endogenous Glucocorticoids and Corticosteroid Drugs” Supporting Information

Sean Coyne,‡ Robert Ghergurovich,‡ Francis Sacco, Francesca Lombardi, Kailey Paar, Jackson DeMartino, Annick A. Kenfack, Tyler M. M. Stack*

Department of Chemistry and Biochemistry, Providence College, 1 Cunningham Square, Providence, Rhode Island 02918, United States

‡These authors contributed equally. *Corresponding Author. Email: tstack@providence.edu

Table of Contents

Table S1. Gene Sequence of Codon Optimized genes in pET28a **.................................... 2**

Table S2. Primer sequences used in the study **.................................... 3**

Table S3. Kinetic parameters of BaDesE and AdDesE at pH 7.2 when fit using the substrate inhibition (SubInh) or Michaelis-Menten (MM) equations **.................................... 3**

Figure S1. SDS-PAGE gel analysis of purified 20-HSDH enzymes. **.................................... 4**

Figure S2. Activity of BaDesE and AdDesE versus DMSO v/v%. **.................................... 4**

Figure S3. ^{1}H NMR spectra (in $CDCl_3$, 400 MHz) of cortisol and the resulting product after reduction by BaDesE. **.................................... 5**

Figure S4. ^{1}H NMR spectra (in $CDCl_3$, 400 MHz) of prednisone and the resulting product after reduction by BaDesE. **.................................... 6**

Figure S5. ^{1}H NMR spectra (in $CDCl_3$, 400 MHz) of prednisolone and the resulting product after reduction by BaDesE. **.................................... 7**

Figure S6. ^{1}H NMR spectra (in $CDCl_3$, 400 MHz) of triamcinolone and the resulting product after reduction by BaDesE. **.................................... 8**

Figure S7. ^{1}H NMR spectra (in $CDCl_3$, 400 MHz) of the resulting reductive products reduction by AdDesE. **.................................... 9**

Figure S8. Rate of reduction of cortisol, prednisone, and prednisolone by BaDesE at pH 7.2. **.................................... 10**

Figure S9. Rate of reduction of cortisol, prednisone, and prednisolone by AdDesE at pH 7.2. **.................................... 10**

Figure S10. Rate of reduction of prednisolone by CsDesC at pH 7.2. **.................................... 11**

Table S1. Gene Sequence of Codon Optimized genes in pET28a

Protein (UniProt Database)	DNA Sequence
Ba20β optimized coding sequence in pET28a	ATGGGCAGCAGCCATCATCATCATCATCACAGCAGCGGCCTGGTGC<br>CGCGCGGCAGCCATATGGAAAATCTGTACTTTCAGAGCGCGGTAGA<br>GTCCTCGCAGATACCGGAAAAAACTGTTGAGCAAATTTTTGACGAA<br>CGTTATCCATTGGATAAATGGAAGGATTCCAATTACTCTATACTTGA<br>CAAATTTAGCATGAGAGGGCGCAAAGGTTTTGTCACCGGCGCGGCG<br>GGCGGTTTGGGACGCAATGCTGCTGCAGCCCTTGCACAGGCCGGTG<br>CCGATGTTGCTCTGGTGGATTTACCTTCCCAAGAAGACAAACTTAC<br>CGAACTTGCAAAGGACATGAGCGAGAGATTTGGCACCAACGTAAT<br>CGCCTTGACATGTGACGTAACAGACACAGTGCAAGTTGCAGAGCTT<br>AAGACTCAGCTGGTGGAGCAGCTGGGAACAGTTGACTTTGCTTTCC<br>TGAATGCGGGCGTGAACGTTCCAGGCGACGATCAGGACGCGACAG<br>AAGAGGTGTGGACCCGAACAATTAACATTAACCTGAATGGTACGTA<br>CCGTACCGGGCGCATTGCACACGAGATTATGCGTGAGCATGGGCAT<br>GGCGGATCACTTATTTTTACTGCAAGTCTTTCGGGCCACAACGCCA<br>ACTACATGATGGGCAGCCCGACTCCAGTTAACGCATATGGGGCAAC<br>AAAAGCCGCGATAATGGAACATTCTCGATATCTGGCTGCTGCCCTC<br>GCAAAAGATGGTATCCGTTCTAACACGATTTCACCAGGTTATGTTT<br>GGAGCGGCATCTTCAACGGACGGATTGATATGCCGGGCCATGATGC<br>TATGCTTGAAGTGGTTCCGATGCACCGATTTGGTACTAATGATGAA<br>ATAGCCAGTACAGTTCTGTTTCTGGCGAGCGATGCATCATCCTACGT<br>AACTGGTACGGATATTCGAGTGGATGGTGGGTATTCAGTTTTCTGA
Ad20β optimized coding sequence in pET28a	ATGGGCAGCAGCCATCATCATCATCATCACAGCAGCGGCCTGGTGC<br>CGCGCGGCAGCCATATGGAAAATTTATATTTCCAAAGCGCTGAAGA<br>ATTTTACGCCGTGTATCCGAAAGATGCTTGGAAAGACCCCAGCTAC<br>CGTTTGATGGATAAATTTTCCCTTAAAGGCAAAAAGGGTTTTGTTAC<br>GGGCGGTGGTGGCGGTATAGGGCGTAACACTGCCGCGGCATGGGC<br>AGAAGCCGGAGCGGACGTTGCTTTGGTAGATATTCCAGCGTCAAAA<br>GACCGTCTGGAACCACTGGCTAAAGAGATGTCGGAAAGATATGGC<br>GTAAAAGTGGTTCCCCTGTATTGTGACGTGTCTGATGAAGAGCAGG<br>TGAACGCACTCAAAGATGATCTGATCCGTGAGTTAGGCACAGTTGA<br>CTTTGCGCATATTAATGCCGGCGTGTGTTTGATGGGTGATGACGTG<br>GATGTCCCCTATTCTACCTGGAAGAAAGTAATTGATATTGACCTTA<br>ACGGTGCCTTTATGACAGCACAGGTTGCGCAGCAAATTATGCGGGA<br>GCATAATCATGGTGGTAGTATCATAATGACCTCATCTCTTTCAGGCT<br>ATAATGCGAACTTCATTGGTGGCGGCCCTTCACCGGTTTGCGCCTAC<br>GGTACAGCTAAAGCAGGAATTTCGCAGCTTGCGCGCTACATGGGGG<br>CGGGACTGGCCCCCTATGGAATACGAGTGAATACCATATCTCCGGG<br>ATATATTTGGAGTGGTATCCACGAAGGTGTTATGGATAAGGCGGGC<br>CATGATATGTGCCTGGAAGTGGTACCGATCAAGCGTTTTGGGCGTA<br>CCGATGAATTACAGGGCGTAATGCTGTTCCTGGCATCCGAAGCCAG<br>TAGCTACATAACGGGCATTAACATTCCTGTTGATGGTGGATATTCA<br>ATATTTTGA

Table S2. Primer sequences used in the study

Primer Name	Primer Sequence
B0NC68_fwd	5’ ctggtgccgcgcggcagccatatgagacaattatttgttacttc 3’
B0NC68_rev	5’ agtggtggtggtggtggtgcctattcgtccatcttaattacg 3’

Table S3. Kinetic parameters of BaDesE and AdDesE at pH 7.2 when fit using the substrate inhibition (SubInh) or Michaelis-Menten (MM) equations

Substrate	Enzyme	Equation	k_{cat} (s^{-1})	k_{cat}/K_m ($M^{-1}s^{-1}$)[a]	K_m (μM)[a]	K_I (μM)
Cortisol	BaDesE	SubInh	3.6 ± 0.2	(3.2 ± 0.5) × 10^6	1.1 ± 0.2	170 ± 30
Cortisol	BaDesE	MM	2.3 ± 0.3	{7 ± 8} × 10^6	{0.3 ± 0.4}	N.A.
Prednisone	BaDesE	SubInh	10. ± 8	(1.4 ± 0.6) × 10^6	7 ± 7	10 ± 10
Prednisone	BaDesE	MM	2.1 ± 0.6	{4 ± 7} × 10^6	{0.5 ± 1.0}	N.A.
Prednisolone	BaDesE	SubInh	9 ± 1	(2.0 ± 0.6) × 10^6	4 ± 1	160 ± 70
Prednisolone	BaDesE	MM	5.0 ± 0.7	(3.5 ± 2.7) × 10^6	1.5 ± 1.2	N.A.
Cortisol	AdDesE	SubInh	0.065 ± 0.008	(6 ± 1) × 10^3	11 ± 2	300 ± 100
Cortisol	AdDesE	MM	0.044 ± 0.003	(1.0 ± 0.3) × 10^4	5 ± 2	N.A.
Prednisone	AdDesE	SubInh	0.116 ± 0.009	(5 ± 1) × 10^4	2.5 ± 0.6	80 ± 20
Prednisone	AdDesE	MM	0.07 ± 0.01	{1 ± 2} × 10^5	{0.7 ± 1.6}	N.A.
Prednisolone	AdDesE	SubInh	0.052 ± 0.004	(4.8 ± 0.7) × 10^3	11 ± 2	500 ± 200
Prednisolone	AdDesE	MM	0.041 ± 0.002	(6 ± 1) × 10^3	6 ± 1	N.A.

N.A. not applicable

a) Values in curly braces have standard errors larger than the determined constants

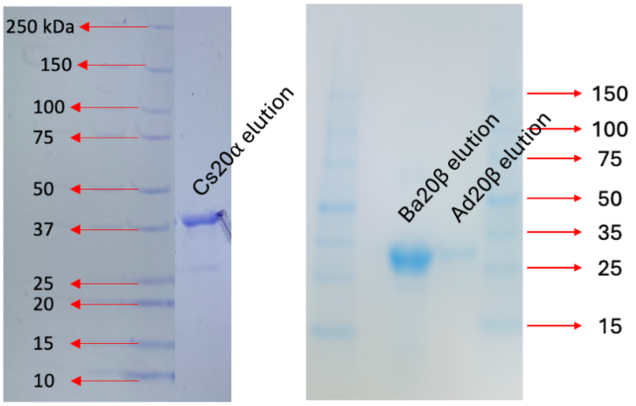

Figure S1. SDS-PAGE gel analysis of purified 20-HSDH enzymes. The ladder markers are in kDa. The expected sizes of Cs20α, Ba20β, and Ad20β are 40.7 kDa, 34.7 kDa, and 33.3 kDa, respectively.

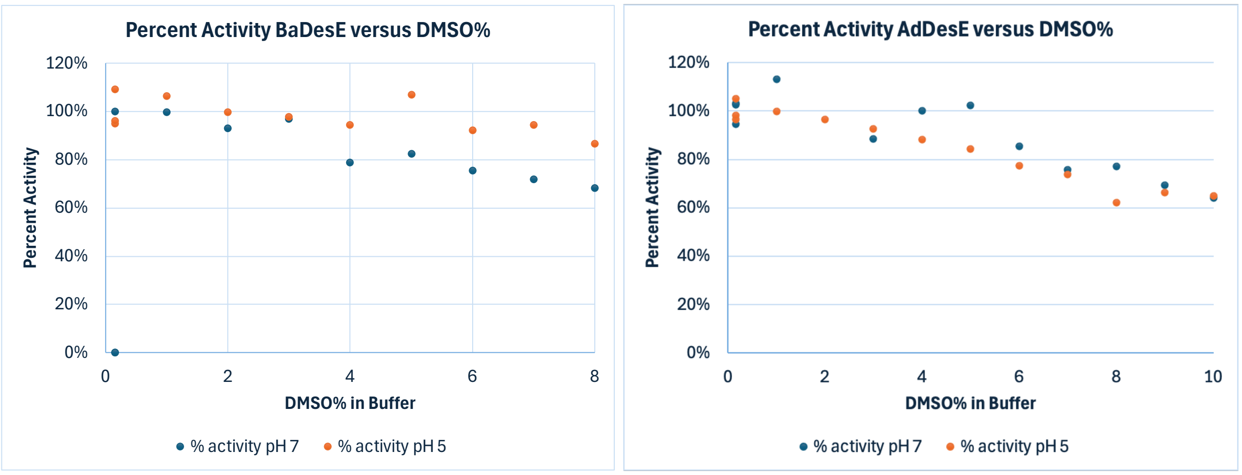

Figure S2. Activity of BaDesE and AdDesE versus DMSO v/v%.

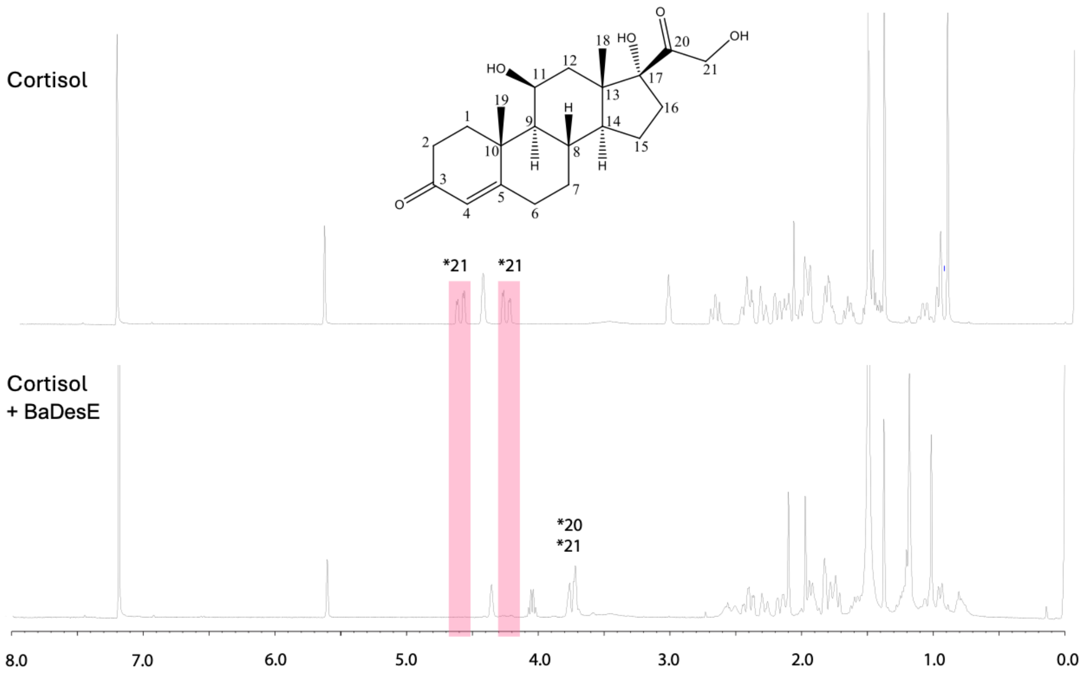

Figure S3. ^{1}H NMR spectra (in $CDCl_3$, 400 MHz) of cortisol and the resulting product after reduction by BaDesE. The ^{1}H peaks corresponding to the C21 hydroxymethylene protons shift and a new peak representative of C20 appear.

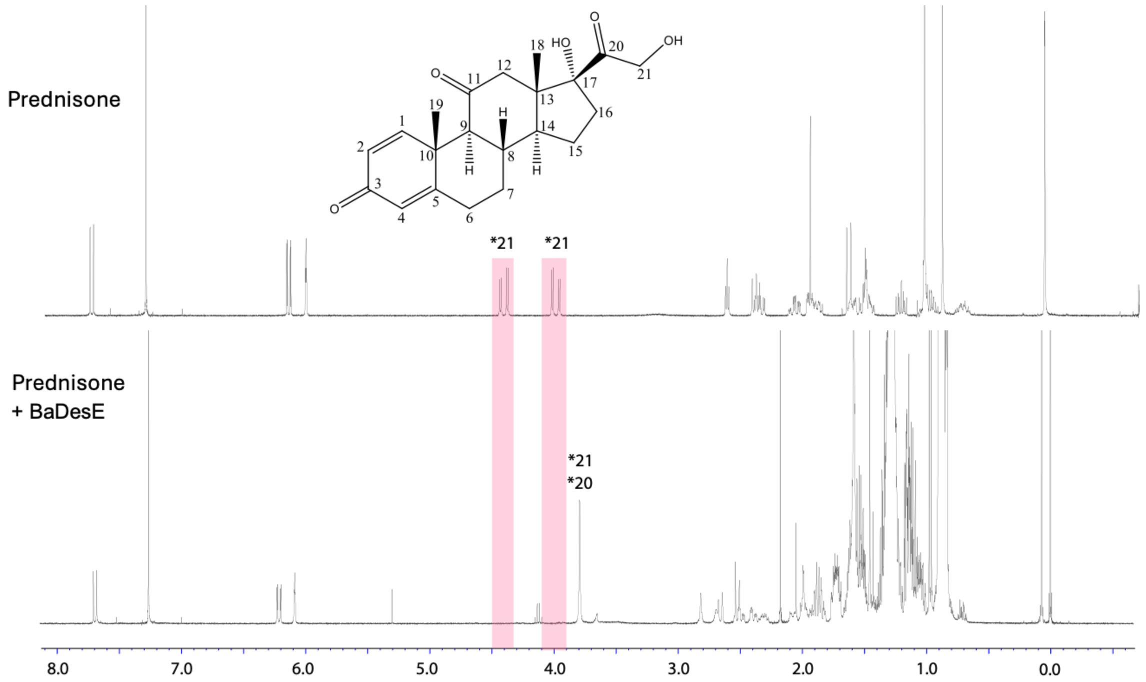

Figure S4. ^{1}H NMR spectra (in $CDCl_3$, 400 MHz) of prednisone and the resulting product after reduction by BaDesE. The ^{1}H peaks corresponding to the C21 hydroxymethylene protons shift, and a new peak representative of C20 appears.

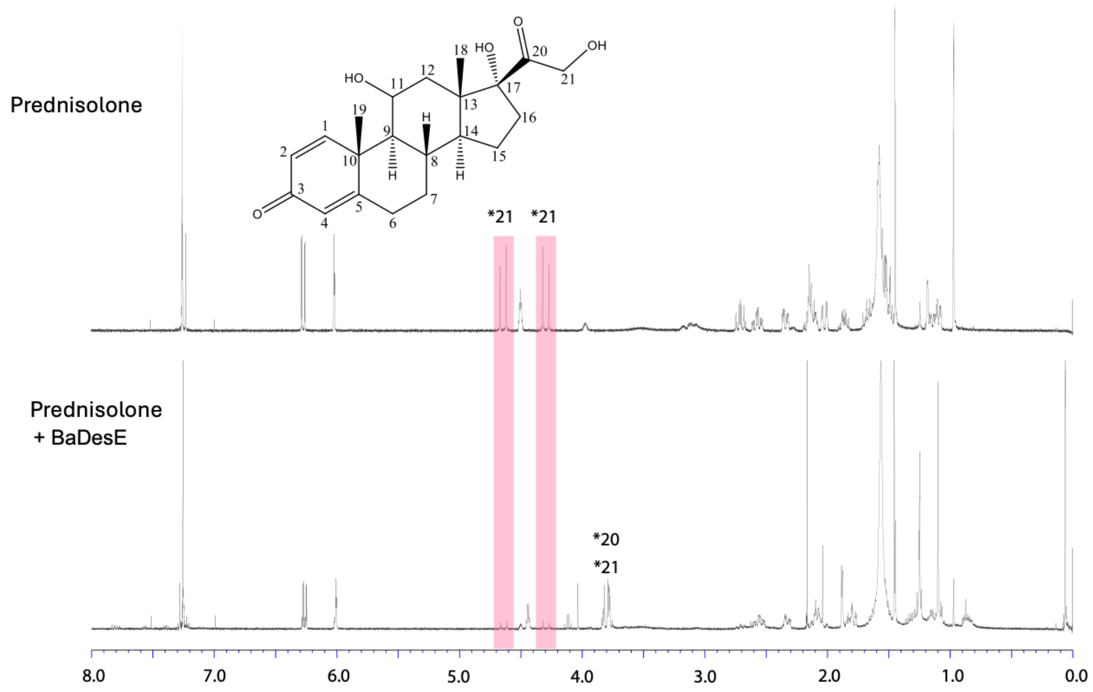

Figure S5. ^{1}H NMR spectra (in $CDCl_3$, 400 MHz) of prednisolone and the resulting product after reduction by BaDesE. The ^{1}H peaks corresponding to the C21 hydroxymethylene protons shift, and a new peak representative of C20 appears.

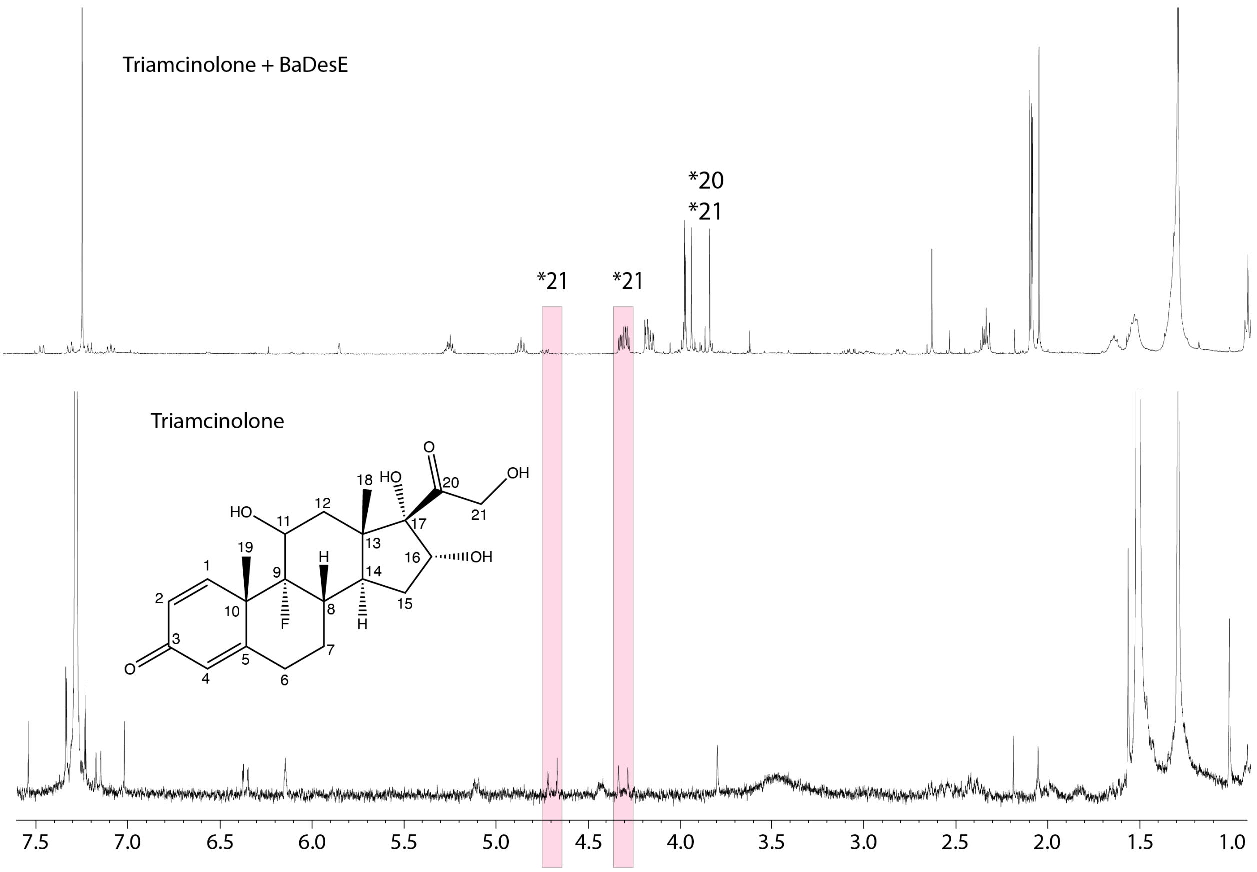

Figure S6. ^{1}H NMR spectra (in $CDCl_3$, 400 MHz) of triamcinolone and the resulting product after reduction by BaDesE. ^{1}H peaks corresponding to the C21 hydroxymethylene protons shift, and a new peak representative of C20 appears.

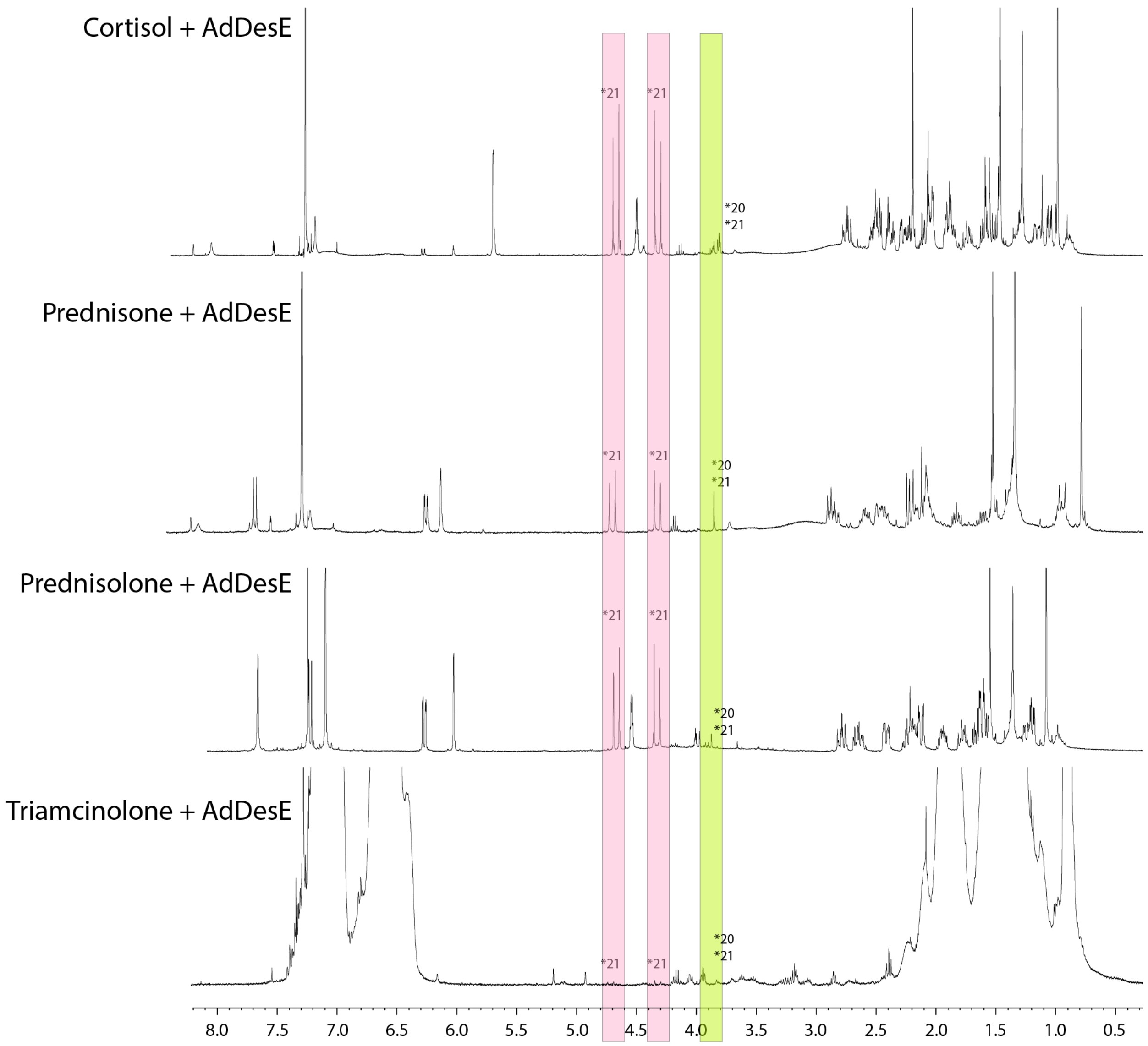

Figure S7. ^{1}H NMR spectra (in $CDCl_3$, 400 MHz) of the resulting reductive products reduction by AdDesE. Only partial conversion was observed. ^{1}H peaks corresponding to the C21 hydroxymethylene protons shift, and a new peak representative of C20 appears.

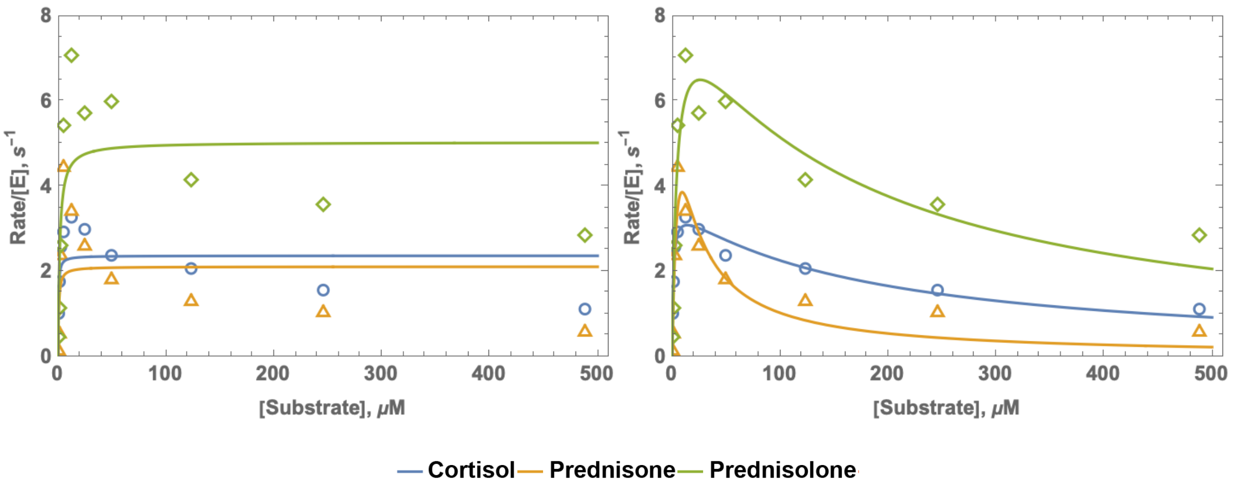

Figure S8. Rate of reduction of cortisol, prednisone, and prednisolone by BaDesE at pH 7.2. These are fit by either the modified Michaelis-Menten equation (left) or the substrate inhibition equation (right), as described in the Methods section. The determined rate constants are reported in **Table S3**.

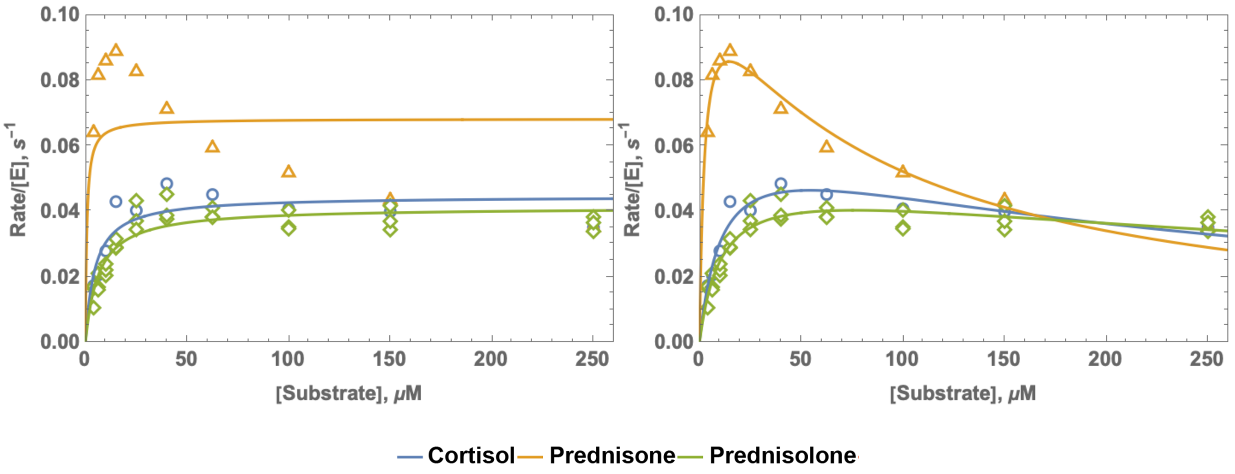

Figure S9. Rate of reduction of cortisol, prednisone, and prednisolone by AdDesE at pH 7.2. These are fit by either the modified Michaelis-Menten equation (left) or the substrate inhibition equation (right), as described in the Methods section. The determined rate constants are reported in **Table S3**.

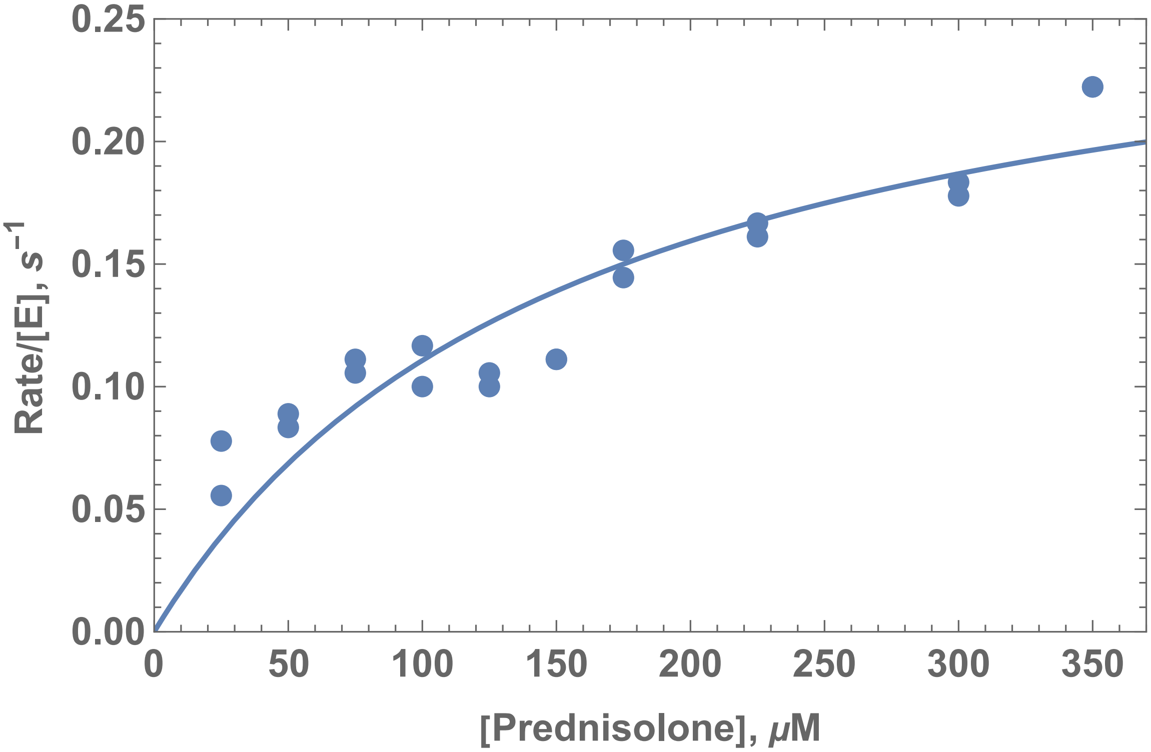

Figure S10. Rate of reduction of prednisolone by CsDesC at pH 7.2. The determined kinetic constants are: $k_{cat}/K_M = (1.8 \pm 0.4) \times 10^3$ M^{-1} s^{-1}, $k_{cat} = 0.28 \pm 0.05$ s^{-1}, and $K_M = 160 \pm 40$ μM.
